# M﻿ultimodal intrinsic speckle-tracking (MIST) to extract images of rapidly-varying diffuse X-ray dark-field

**DOI:** 10.1038/s41598-023-31574-z

**Published:** 2023-04-03

**Authors:** Samantha J. Alloo, Kaye S. Morgan, David M. Paganin, Konstantin M. Pavlov

**Affiliations:** 1 School of Physical and Chemical Sciences, University of Canterbury, Christchurch, New Zealand; 2 School of Physics and Astronomy, Monash University, Clayton, VIC Australia; 3 School of Science and Technology, University of New England, Armidale, NSW Australia

**Keywords:** Applied physics, Optical physics, Techniques and instrumentation

## Abstract

Speckle-based phase-contrast X-ray imaging (SB-PCXI) can reconstruct high-resolution images of weakly-attenuating materials that would otherwise be indistinguishable in conventional attenuation-based X-ray imaging. The experimental setup of SB-PCXI requires only a sufficiently coherent X-ray source and spatially random mask, positioned between the source and detector. The technique can extract sample information at length scales smaller than the imaging system’s spatial resolution; this enables multimodal signal reconstruction. “Multimodal Intrinsic Speckle-Tracking” (MIST) is a rapid and deterministic formalism derived from the paraxial-optics form of the Fokker–Planck equation. MIST simultaneously extracts attenuation, refraction, and small-angle scattering (diffusive dark-field) signals from a sample and is more computationally efficient compared to alternative speckle-tracking approaches. Hitherto, variants of MIST have assumed the diffusive dark-field signal to be spatially slowly varying. Although successful, these approaches have been unable to well-describe unresolved sample microstructure whose statistical form is not spatially slowly varying. Here, we extend the MIST formalism such that this restriction is removed, in terms of a sample’s rotationally-isotropic diffusive dark-field signal. We reconstruct multimodal signals of two samples, each with distinct X-ray attenuation and scattering properties. The reconstructed diffusive dark-field signals have superior image quality—as measured by the naturalness image quality evaluator, signal-to-noise ratio, and azimuthally averaged power-spectrum—compared to our previous approaches which assume the diffusive dark-field to be a slowly varying function of transverse position. Our generalisation may assist increased adoption of SB-PCXI in applications such as engineering and biomedical disciplines, forestry, and palaeontology, and is anticipated to aid the development of speckle-based diffusive dark-field tensor tomography.

X-rays are high-energy ionizing electromagnetic radiation that can penetrate and pass through objects. Since being serendipitously discovered in the late 19th century by Röntgen^1^, X-rays have been employed in numerous disciplines. X-rays are modified when passing through an object, by means of attenuation and phase-shifts (refraction), making them useful in imaging applications as the modified X-ray wavefield contains detailed sample information. Currently, in conventional medical imaging applications, only X-ray attenuation is considered when generating image contrast. This works well when imaging objects whose composite material’s X-ray attenuation characteristics are considerably different as the generated image contrast is high, for example, for bones and soft-tissue in radiography. However, attenuation contrast is insufficient when imaging objects whose composite materials attenuate the X-ray beam similarly, for example, adipose and glandular tissues in mammography. Such weakly-attenuating materials can be more readily distinguished when X-ray refraction is also utilised to generate image contrast as typically these materials refract X-rays more dissimilarly. X-ray imaging techniques that utilise both X-ray attenuation and refraction to generate image contrast are called phase-contrast X-ray imaging (PCXI)^2^ techniques, where the X-ray attenuation and refraction can be described by the material’s imaginary and real components of its refractive index, ${n({\textbf{r}}')=1 - \delta ({\textbf{r}}') + i \beta ({\textbf{r}}')}$, respectively, where ${\textbf{r}}'$ is the three-dimensional position vector. The superiority of PCXI techniques over attenuation-based imaging has already been proven in multiple disciplines. Such studies have been performed using synchrotron and conventional laboratory X-ray sources for applications in several fields, including biomedical^3–6^, agricultural and food sciences^7^, palaeontology^8,9^, and materials science^10,11^.

PCXI techniques convert sample-imposed phase effects into measurable detector intensity differences and there are various methods to do so in the current research literature. Propagation-based^12,13^ imaging is the experimentally-simplest technique, achieved by illuminating the sample with a spatially coherent beam and positioning the detector a sufficient distance downstream such that the beam self-interferes at locations of phase differences (e.g. at edges). This approach eliminates the need for complex optics, however, it does require sufficiently large sample-to-detector distances and has stringent coherence requirements. In the aim of decreasing coherence requirements and increasing phase sensitivity, PCXI techniques that introduce suitable optical elements into the experimental setup have also been developed, for example, via grating-interferometry^14^, analyser-based imaging^15^, grid-based imaging^16,17^, edge-illumination imaging^18^, and speckle-based (SB) imaging^19,20^. Grating-interferometry^14^ measures an object’s differential phase by positioning two periodic gratings along the set-up’s optical axis. The second grating is placed such that moiré fringes are produced, and the sample-induced modifications to these fringes are utilised to reconstruct sample phase information. An analyser crystal, for example, Si, is introduced in analyser-based imaging^15^, and the analysis of local rocking curves of the transmitted and/or diffracted X-ray beam allows for sample attenuation and refraction information to be extracted. Grid-based imaging^16,17,21^ is a single-exposure technique that measures the sample-induced vertical and horizontal shift and blur of a two-dimensional periodic reference pattern. In edge-illumination imaging^18^, one aperture is used to collimate the incident X-ray beam, and a second one is positioned on the detector so that only the edge of each pixel is illuminated, and hence changes in phase or scattering change the intensity seen by each pixel.

The resolution of signal reconstruction in PCXI techniques is predominantly restricted by the detector’s pixel size. Sample structures at length scales smaller than the spatial resolution of the imaging system induce local small-angle X-ray scattering (SAXS). Some PCXI techniques can provide information regarding these unresolved sample microstructures through diffusive dark-field (DF) imaging as DF image contrast is formed from the mechanism of SAXS. PCXI techniques that reconstruct the coherent, phase-contrast (PC), and diffuse, DF, flows of an X-ray wavefield are often termed “multimodal”, in the sense that they can extract multiple signals. Grating interferometry^22^, analyser-based imaging^23,24^, grid-based imaging^21^, edge-illumination imaging^25^, and propagation-based imaging^26,27^ are examples of PCXI techniques that are sensitive to local SAXS, and can therefore reconstruct a sample’s DF signal.

Realised just a decade ago^19,20^, SB-PCXI is a particularly appealing multimodal technique as it is experimentally simple, cost-effective, and radiation dose-efficient. A speckle pattern is generated when a sufficiently coherent wavefield propagates through a membrane with random refractive index fluctuations^28^. A speckle pattern, in the context of SB-PCXI, is used as an X-ray wavefront marker whose subsequent modification is used to measure sample-induced speckle modulations, e.g., transverse spatial shifts, attenuation, and blurring. The SB-PCXI experimental setup consists of an X-ray source, speckle-generating mask, sample, and detector system, positioned some finite distance downstream from the sample, as shown in Fig. 1. In this work, we consider a filtered synchrotron X-ray source that produces a monochromatic, paraxial wavefield having a high degree of both spatial and temporal coherence. The X-ray wavefield is randomly modulated by propagation through the speckle-generating mask (e.g., conventional sandpaper) and is then registered by the position-sensitive detector positioned downstream. We highlight two particularly attractive features of SB-PCXI. Firstly, the speckle-generating mask can be any spatially-random medium. This removes the restriction of needing precisely-manufactured optical elements, making the experimental setup both easy to implement and flexible. Secondly, SB-PCXI requires relatively low spatial and temporal coherence^29^.

The inverse problem^30^ of SB-PCXI involves reconstructing a sample’s multimodal signals given suitable reference-speckle and sample-reference-speckle intensity images. The reference-speckle images resemble the composition of the mask and the sample-reference-speckle images are captured when a sample is placed into this reference-speckle field. This reference-speckle pattern is modified, depending on the refractive properties of the sample, and these speckle modifications are used to reconstruct sample information. Transverse speckle shifts are associated with the PC signal, whereas speckle blurring, or reduction in visibility, is correlated to the sample’s DF signal. There are two distinct approaches in the research literature to solve the multimodal inverse SB-PCXI problem, namely, extrinsic and intrinsic speckle-tracking approaches. X-ray speckle-vector tracking^19^ (XSVT), mixed XSVT approaches^31,32^, X-ray speckle-scanning^33,34^ (XSS), and unified modulated pattern analysis^35^ (UMPA) are examples of extrinsic approaches that reconstruct multimodal signals using iterative pixel-wise methods on SB-PCXI data acquired at multiple mask positions. Although these extrinsic approaches can reconstruct multimodal signals from just two mask positions, see Fig. 2b,c in Ref. ^36^, they typically require several, approximately ten, to achieve reasonable resolution and reduce noise. The first demonstrations of XSS used local cross-correlation on a single reference-speckle and single sample-reference-speckle image to evaluate sample-induced changes^19,20^. However, to improve the spatial resolution of the retrieved image, XSS evolved from using a “single set” of data to using multiple speckle mask positions. In essence, XSVT, mixed XSVT, and XSS approaches perform a cross-correlation analysis, between the reference-speckle and sample-reference-speckle images. However, in XSS, unlike XSVT or mixed XSVT, the speckle pattern needs to be shifted at a known constant step size throughout the entire scan. This is because the cross-correlation analysis is performed pixel-wise (pixel-per-pixel) in XSS, compared to XSVT and mixed XSVT which use larger analysis windows. Evidently, XSS has higher resolution, however, it is also more sensitive to experimental instabilities. UMPA proposes a different methodology, based on least-squares minimisation between a model and the measurement of the sample-reference-speckle pattern across all mask positions. XSVT, mixed XSVT, and UMPA have the advantage of a relatively short image acquisition time compared to XSS^33,34^ techniques. An extensive evaluation and description of the above-mentioned extrinsic speckle-tracking approaches is provided in Ref. ^29^. The SB-PCXI inverse problem was reconceptualised in 2018 when Paganin *et al.*^37^ proposed a geometric-flow approach to reconstruct a sample’s PC signal using a single set (one reference-speckle image and one sample-reference-speckle image) of SB-PCXI intensity data; this was the first realisation of so-called intrinsic speckle-tracking, since it does not explicitly track individual speckles, but rather solves a partial differential equation formulated at the whole-of-image level. This intrinsic speckle-tracking geometric-flow formalism^37^ was then combined with a Fokker–Planck-type^38,39^ generalisation of the transport-of-intensity equation^40^ of paraxial wave optics to allow for multimodal intrinsic signal extraction^36^, which was named “Multimodal Intrinsic Speckle-Tracking” (MIST). The Fokker–Planck^41^ expression is based on local energy conservation, and it considers transverse radiation flows as a combination of coherent and diffusive effects; these effects for the case of multimodal signal extraction align with the PC and DF signals, respectively. MIST is less computationally expensive than the alternative extrinsic approaches^19,20,31–35^. This means that MIST can be used to rapidly reconstruct tomographic data^42^ of both the PC and DF signals, therefore, to provide complementary three-dimensional sample information that is inaccessible in single-projection imaging.

MIST was first developed^36^ under three key assumptions: (a) the sample is a pure phase-object, such that X-ray attenuation can be neglected, (b) the unresolved sample microstructure diffusely scatters the X-ray beam in a rotationally-isotropic manner, and (c) the sample’s DF signal is a slowly varying function of transverse position. The first assumption, (a), was relaxed in our most recent work where we presented the case of attenuating materials having rotationally-isotropic position-dependent diffuse scatter^42^. Here, we should highlight that we have also performed isotropic DF computed-tomography (CT) using MIST^42^, as the reconstructed two-dimensional DF signals had a sufficiently high signal-to-noise ratio (SNR) and spatial resolution such that they could be used in standard CT reconstruction algorithms, such as filtered back-projection^43^. Assumption (b) was relaxed when we considered anisotropically-scattering attenuating materials^44^, such that directional DF^45,46^ signals could be obtained. Assumption (c), which considers the sample’s DF signal to be spatially slowly varying, is a condition that has remained in all of the MIST approaches to date. Although both Pavlov *et al.*^36^ and Alloo *et al.*^42^ have demonstrated that this approach to MIST is capable of reconstructing images with a high spatial resolution, which is at least comparable to the alternative extrinsic approaches, our recent investigations have found that this approximation may break down at sharp interfaces. This assumption may hinder the full potential of previous MIST approaches. In particular, samples whose microstructure autocorrelation function varies rapidly as a function of transverse position within the sample may breach the domain of applicability of all previous MIST approaches. In the present paper, we generalise MIST to alleviate this restriction, thereby broadening its domain of utility. Several studies have verified the broad applicability and importance of DF imaging^34,47,48^, with a significant focus on biomedical clinical applications^49^, e.g., using DF imaging for early-stage diagnosis of lung diseases such as fibrosis^50,51^, pneumothorax^52^, emphysema^53^, and breast cancer^26^. Our improved MIST approach might provide an alternative experimentally versatile, low-dose imaging technique that can reconstruct high-resolution multimodal signals in two- and three-dimensions, with CT achieved as shown with the earlier variant of MIST^42^.

This paper progresses as follows. First, we theoretically develop the new generalised MIST approach, deriving an analytical solution for a sample’s phase-shift (PC signal) and effective diffusion coefficient (DF signal). Numerical stabilisation techniques are then discussed, before applying the approach to synchrotron SB-PCXI data of two samples that have different X-ray attenuation and scattering characteristics. Moreover, we first consider a sample that is weakly-attenuating (almost a pure phase-object), and then a more attenuating object to investigate the breadth of applicability of our approach. We then compare this new approach, qualitatively and quantitatively, to both of the published rotationally-isotropic MIST approaches^36,42^: (a) Pavlov *et al.*’s approach^36^, which neglects X-ray attenuation, and (b) Alloo *et al.*’s approach^42^, which considers it. Note that both previous approaches approximate the DF to be spatially slowly varying. The paper is finished by discussing potential future research avenues.

**Figure 1:**
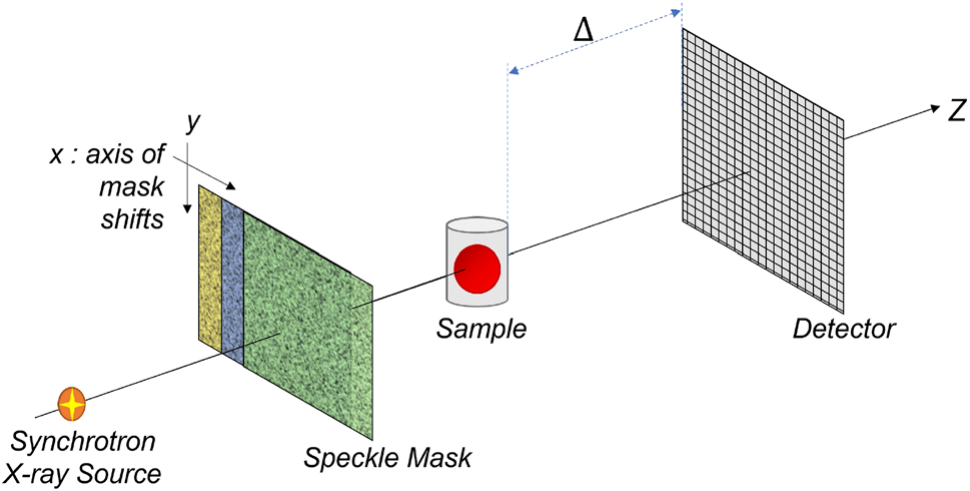
Experimental setup of speckle-based phase-contrast X-ray imaging using a synchrotron X-ray source.

## Theoretical derivation of updated MIST approach

The SB-PCXI form of the Fokker–Planck equation models the coherent and diffusive flows of X-rays in the case of a phase-object, as described by its phase-shift, $\phi _{\text {ob}}({\textbf {r}})$, and effective diffusion coefficient, $D_{\text {eff, Phase}}({\textbf {r}};\Delta )$, respectively. The SB-PCXI Fokker–Planck equation is derived assuming the reference-speckle field is spatially well-resolved, and that this field, alongside the speckle field in the presence of the sample, obeys a Fokker–Planck^41^ extension of the geometric-flow formalism for speckle tracking^37^. The Fokker–Planck equation in this instance is^38^

1 $$\begin{aligned} I_R({\textbf {r}})-I_S({\textbf {r}}) = \frac{\Delta }{k}\nabla _{\perp }\cdot \left[ I_{R}({\textbf {r}})\nabla _{\perp }\phi _{\text {ob}}({\textbf {r}})\right] -\Delta \nabla _{\perp }^{2}\left[ D_{\text {eff, Phase}}({\textbf {r}};\Delta )I_{R}({\textbf {r}})\right] , \end{aligned}$$

where $I_R({\textbf {r}})$ and $I_S({\textbf {r}})$ denote the reference-speckle and sample-reference-speckle intensities, respectively, ${\textbf {r}}\equiv (x,y)$ denotes Cartesian coordinates in planes perpendicular to the optical axis *z*, assuming plane-wave illumination, $\Delta$ is the sample-to-detector distance, *k* is the wavenumber, $\nabla _{\perp } = (\partial /\partial x, \partial /\partial y)$ is the transverse gradient operator in the (*x*, *y*) plane, and $\nabla _{\perp }^{2}$ is the transverse Laplacian operator. The reference-speckle intensity, $I_R({\textbf {r}})$, is obtained with the speckle mask in and the object out of the X-ray beam, and the sample-reference-speckle intensity, $I_S({\textbf {r}})$, is obtained by placing a phase- and SAXS-inducing object into the speckle-modulated X-ray beam. In previous rotationally-isotropic MIST approaches^36,42^, the Fokker–Planck equation has been simplified (for phase-objects) to a linear equation in terms of the effective diffusion coefficient and the Laplacian of the phase-shift. This simplification resulted by assuming the effective diffusion coefficient to be spatially slowly varying, such that various second-order terms could be neglected. In the present work, we do not make this assumption, and instead, the effective diffusion coefficient can accurately describe the diffuse scattering signal from unresolved sample microstructure for which the autocorrelation function can be a rapidly varying function of transverse position.

The present approach begins by expanding the coherent flow term, namely the first term on the right side of the preceding equation, into its two components that describe the lensing and prism-like effects^38^:

2 $$\begin{aligned} \frac{\Delta }{k}\nabla _{\perp }\cdot \left[ I_{R}({\textbf {r}})\nabla _{\perp }\phi _{\text {ob}}({\textbf {r}})\right] = \frac{\Delta }{k}\left[ I_{R}({\textbf {r}})\nabla _{\perp }^2\phi _{\text {ob}}({\textbf {r}}) + \nabla _{\perp }I_{R}({\textbf {r}})\cdot \nabla _{\perp }\phi _{\text {ob}}({\textbf {r}})\right] . \end{aligned}$$

Following the approximation described in Pavlov *et al.*^36^, we neglect the scalar product of the gradient of the random rapidly-varying wavefield intensity $I_{R}({\textbf {r}})$ with the more slowly changing gradient of the wavefield phase $\phi _{\text {ob}}({\textbf {r}})$, namely, we assume that

3 $$\begin{aligned} \frac{\Delta }{k}\nabla _{\perp }\cdot \left[ I_{R}({\textbf {r}})\nabla _{\perp }\phi _{\text {ob}}({\textbf {r}})\right] \approx \frac{\Delta }{k} I_{R}({\textbf {r}})\nabla _{\perp }^2\phi _{\text {ob}}({\textbf {r}}). \end{aligned}$$

Hence equation (1) becomes (cf. Ref. ^36^)

4 $$\begin{aligned} I_R({\textbf {r}})-I_S({\textbf {r}}) = \frac{\Delta }{k}I_{R}({\textbf {r}})\nabla _{\perp }^2\phi _{\text {ob}}({\textbf {r}})-\Delta \nabla _{\perp }^{2}\left[ D_{\text {eff, Phase}}({\textbf {r}};\Delta )I_{R}({\textbf {r}})\right] . \end{aligned}$$

The associated inverse problem of multimodal SB-PCXI, in this work, involves solving equation (4) for $D_{\text {eff, Phase}}({\textbf {r}};\Delta )$ and $\phi _{\text {ob}}({\textbf {r}})$, given the SB-PCXI intensity data, $I_R({\textbf {r}})$ and $I_S({\textbf {r}})$. We expand the final term on the right side of equation (4) using the vector identity $\nabla _{\perp }^2(AB)=A\nabla _{\perp }^2B+B\nabla _{\perp }^2A+2\nabla _{\perp }A\cdot \nabla _{\perp }B$ ^54^ for scalar functions *A* and *B* to give:

5 $$\begin{aligned}&\frac{1}{\Delta }\left[ I_R({\textbf {r}})-I_S({\textbf {r}})\right] = I_{R}({\textbf {r}})\nabla _{\perp }^2\left[ \frac{1}{k}\phi _{\text {ob}}({\textbf {r}})-D_{\text {eff, Phase}}({\textbf {r}};\Delta )\right] -D_{\text {eff, Phase}}({\textbf {r}};\Delta )\nabla _{\perp }^{2}I_{R}({\textbf {r}})\nonumber \\&\quad -2D_{\text {eff, Phase}}^x({\textbf {r}};\Delta )I_{R}^x({\textbf {r}})-2D_{\text {eff, Phase}}^y({\textbf {r}};\Delta )I_{R}^y({\textbf {r}}). \end{aligned}$$

This is a linear equation in terms of four unknowns—namely, $\nabla _{\perp }^2\left[ \frac{1}{k}\phi _{\text {ob}}({\textbf {r}})-D_{\text {eff, Phase}}({\textbf {r}};\Delta )\right]$, $D_{\text {eff, Phase}}({\textbf {r}};\Delta )$, $D_{\text {eff, Phase}}^x({\textbf {r}};\Delta )$, and $D_{\text {eff, Phase}}^y({\textbf {r}};\Delta )$—where the partial derivatives in the spatial coordinates *x* and *y* are denoted with their respective superscripts. These can then be employed to reconstruct the sample’s true phase-shifts and effective diffusion coefficient. To obtain unique solutions for four unknown variables we require four equations; to do this, four unique forms of equation (5) can be generated by taking four independent measurements of SB-PCXI data, where one SB-PCXI data “set” consists of an $I_R({\textbf {r}})$ and $I_S({\textbf {r}})$ pair. This can be achieved by, for example, transversely shifting the mask to generate a new reference-speckle pattern, and subsequently sample-reference-speckle pattern. Let subscript *n* denote an independent SB-PCXI data set, then four independent measurements of $I_R({\textbf {r}})$ and $I_S({\textbf {r}})$ will give:

6 $$\begin{aligned}&\frac{1}{\Delta }\left[ I_{R_{n}}({\textbf {r}})-I_{S_{n}}({\textbf {r}})\right] = I_{R_{n}}({\textbf {r}})\nabla _{\perp }^2\left[ \frac{1}{k}\phi _{\text {ob}}({\textbf {r}})-D_{\text {eff, Phase}}({\textbf {r}};\Delta )\right] -D_{\text {eff, Phase}}({\textbf {r}};\Delta )\nabla _{\perp }^{2}I_{R_{n}}({\textbf {r}})\nonumber \\&\quad -2D_{\text {eff, Phase}}^x({\textbf {r}};\Delta )I_{R_{n}}^x({\textbf {r}})-2D_{\text {eff, Phase}}^y({\textbf {r}};\Delta )I_{R_{n}}^y({\textbf {r}}),\ n = 1,\ 2,\ 3,\ \text {and } 4. \end{aligned}$$

Equation (6) gives a linear system that can be solved using, for example, Gaussian-elimination. Here, it is important to highlight that previous MIST approaches for rotationally-isotropic diffuse scatter^36,42^ require SB-PCXI data from just two mask positions. Within the present method, we require four mask positions since we extract two additional quantities, namely $D_{\text {eff, Phase}}^x({\textbf {r}};\Delta )$ and $D_{\text {eff, Phase}}^y({\textbf {r}};\Delta )$. These terms are small for samples whose unresolved microstructure can be considered to have an autocorrelation that is a slowly varying function of transverse position. However, these terms are significant in the contrary case where the sample’s unresolved-microstructure autocorrelation functions are spatially rapidly varying. These terms are also important to correctly reconstruct sample edges.

A sample’s true effective diffusion coefficient can be reconstructed by aggregating the three extracted quantities, $D_{\text {eff, Phase}}({\textbf {r}};\Delta )$, $D_{\text {eff, Phase}}^x({\textbf {r}};\Delta )$, and $D_{\text {eff, Phase}}^y({\textbf {r}};\Delta )$. We employ a method adopted in various differential imaging techniques^17,55–57^ whereby a two-dimensional function, *g*(*x*, *y*), can be calculated from its two spatial derivatives, $g^x(x,y)$ and $g^y(x,y)$, using the identity:

7 $$\begin{aligned} g(x,y) = \mathscr {F}^{-1}\left[ \frac{\mathscr {F}\left( g^x(x,y)+ig^y(x,y)\right) }{ik_x-k_y}\right] . \end{aligned}$$

Here, $\mathscr {F}$ denotes two-dimensional Fourier transformation with respect to *x* and *y*, with $k_x$ and $k_y$ being the corresponding Fourier-space variables, respectively. This expression is unstable near the Fourier-space origin, $(k_x, k_y) = (0, 0)$, hence the solution will diverge at low spatial frequencies unless sufficiently regularised. An effective diffusion coefficient can be calculated by equation (7), using the two calculated spatial derivatives $D_{\text {eff, Phase}}^x({\textbf {r}};\Delta )$ and $D_{\text {eff, Phase}}^y({\textbf {r}};\Delta )$. This solution will be stable at high spatial frequencies, that is, far from the Fourier-space origin, and the opposite is true for the effective diffusion coefficient $D_{\text {eff, Phase}}({\textbf {r}};\Delta )$ that is extracted directly from the system of linear equations (equation (6)). These two solutions can be combined to consider only the stable spatial frequencies in each DF solution. In particular, a true effective diffusion coefficient, $D_{\text {eff, Phase}}^{\text {True}}({\textbf {r}};\Delta )$, can be calculated by implementing the following Fourier-space weighted filtering (cf. Ref. ^58^), where $\rho$ is a cut-off parameter:

8 $$\begin{aligned}{} & {} D_{\text {eff, Phase}}^{\text {True}}({\textbf {r}};\Delta ) = \mathscr {F}^{-1}\left[ e^{-\rho (k_x^2+k_y^2)}\mathscr {F}\left( D_{\text {eff, Phase}}({\textbf {r}};\Delta )\right) \right. \nonumber \\{} & {} \quad \left. + \frac{1-e^{-\rho (k_x^2+k_y^2)}}{ik_x-k_y}\mathscr {F}\left( D_{\text {eff, Phase}}^x({\textbf {r}};\Delta )+iD_{\text {eff, Phase}}^y({\textbf {r}};\Delta )\right) \right] . \end{aligned}$$

This completes our description of the method for reconstructing a phase-object’s true effective diffusion coefficient. The sample-induced phase-shift term, $\phi _{\text {ob}}({\textbf {r}})$, can then be reconstructed by utilising equation (4), via

9 $$\begin{aligned} \phi _{\text {ob}}({\textbf {r}}) = \nabla _{\perp }^{-2}\left[ \frac{k}{\Delta I_{R}({\textbf {r}})}\left( I_R({\textbf {r}})-I_{S}({\textbf {r}})+\Delta \nabla _{\perp }^{2}\left[ D_{\text {eff, Phase}}^{\text {True}}({\textbf {r}};\Delta )I_{R}({\textbf {r}})\right] \right) \right] . \end{aligned}$$

In the above expression,

10 $$\begin{aligned} \nabla _{\perp }^{-2} = -\mathscr {F}^{-1}\frac{1}{k_x^2+k_y^2}\mathscr {F} \end{aligned}$$

is the inverse Laplacian operator, derived using the two-dimensional Fourier derivative theorem^2^. The above PC signal extraction is more numerically stable than utilising the $\nabla _{\perp }^2\left[ \frac{1}{k}\phi _{\text {ob}}({\textbf {r}})-D_{\text {eff, Phase}}({\textbf {r}};\Delta )\right]$ term reconstructed from QR decomposition^59^, as that solution would inherently suffer from the numerical instabilities associated with $D_{\text {eff, Phase}}({\textbf {r}};\Delta )$, $D_{\text {eff, Phase}}^x({\textbf {r}};\Delta )$ and $D_{\text {eff, Phase}}^y({\textbf {r}};\Delta )$, whereas equation (9) considers the stabilised $D_{\text {eff, Phase}}^{\text {True}}({\textbf {r}};\Delta )$.

Up until this point, X-ray attenuation by the sample has been neglected. We now extend our analysis to the case of a weakly-attenuating object and calculate its effective diffusion coefficient. We do this based on a relationship obtained in Alloo *et al.*^42^ (see equations (24) and (18) therein) in which an attenuating-object’s effective diffusion coefficient, $D_{\text {eff, Atten}}^{\text {True}}({\textbf {r}};\Delta )$, can be calculated from the phase-object approximation, $D_{\text {eff, Phase}}^{\text {True}}({\textbf {r}};\Delta )$, using

11 $$\begin{aligned} D_{\text {eff, Atten}}^{\text {True}}({\textbf {r}};\Delta ) = \frac{D_{\text {eff, Phase}}^{\text {True}}({\textbf {r}};\Delta )}{I_{\text {ob}}({\textbf {r}})}. \end{aligned}$$

Above, $I_{\text {ob}}({\textbf {r}})$ is the object’s attenuation term describing the intensity at the exit surface of the sample, $z=0$, after the object has attenuated the incident X-ray beam of unit intensity. To calculate $I_{\text {ob}}({\textbf {r}})$, we consider a single-material object such that the projection approximation^2^ can be written as $\phi _{\text {ob}}({\textbf {r}}) = -k \delta t({\textbf {r}})$ and $I_{\text {ob}}({\textbf {r}}) = \text {exp}\left[ -2k\beta t({\textbf {r}})\right]$, where $t({\textbf {r}})$ is the projected thickness of the object along the direction *z* of the X-rays^60^. Hence

12 $$\begin{aligned} I_{\text {ob}}({\textbf {r}}) = \text {exp}\left[ \frac{2\phi _{\text {ob}}({\textbf {r}})}{\gamma }\right] , \end{aligned}$$

where $\gamma = \delta /\beta$ for the single-material object. This attenuation term, obtained using the phase-shift term from equation (9), can then be used in equation (11) to reconstruct $D_{\text {eff, Atten}}^{\text {True}}({\textbf {r}};\Delta )$. Although in theory this attenuation extraction is restricted to single-material objects, it can be extended to multi-material objects by taking the difference in the refractive index components^61,62^ for composite materials. Furthermore, in a tomographic context, it has been proven that this approximation does not affect the reconstructed attenuation coefficient, $\beta ({\textbf{r}}')$, far away from material interfaces^63^, and hence, this restriction would only be adverse in a sample that has several composite materials with significantly differing attenuation and refraction properties.

## Stabilising the SB-PCXI multimodal inverse problem

The inverse problem^30^ of reconstructing the effective diffusion coefficient requires appropriate numerical regularisation. Here, we apply the common approach^64,65^ of a Tikhonov regularisation^66^, which sufficiently stabilises the signal reconstruction. In its simplest form, a Tikhonov regularisation of the quotient of two functions *A* and *B* can be employed, using

13 $$\begin{aligned} \frac{A}{B} \rightarrow \frac{AB}{B^2+\alpha }, \end{aligned}$$

where $\alpha \ge 0$ is a regularisation parameter whose magnitude is sufficiently small compared to $B^2$.

The theoretical approach in the present paper reconstructs a sample’s multimodal signals by solving a full-rank system of four linear equations, given by equation (6). Although the theoretical minimum is four sets of SB-PCXI data, the numerical stability of the solutions can be improved by utilising SB-PCXI data from more mask positions. Namely, for *N* mask positions, we can generate an over-determined system of *N* linear equations following the form of equation (5). The system generated can then be solved in a least-squares sense using pixel-wise QR decomposition^59^. Tikhonov’s regularisation method can also be applied to an ill-posed least-squares problem (QR factorisation), as described by Zhu^67^. Namely, rather than using QR decomposition to solve the linear system $A\bar{x} = \bar{b}$ for the least-squares solution $\tilde{x}$, QR decomposition can instead be performed on the system $$\begin{pmatrix}A;\alpha I\end{pmatrix}\bar{x}=\begin{pmatrix}\bar{b};0\end{pmatrix},$$ where $N \times M$ is the coefficient matrix *A*, “;” denotes a new row, *I* is the $M \times M$ identity matrix, $\alpha$ is the chosen regularisation parameter, and the right-hand-side vector $\bar{b}$ is filled with zeroes to reach the size of $(N+M) \times 1$. The described Tikhonov-regularised QR decomposition can be used to solve the over-determined system of linear equations for the four unknown variables, $\nabla _{\perp }^2\left[ \frac{1}{k}\phi _{\text {ob}}({\textbf {r}})-D_{\text {eff, Phase}}({\textbf {r}};\Delta )\right]$, $D_{\text {eff, Phase}}({\textbf {r}};\Delta )$, $D_{\text {eff, Phase}}^x({\textbf {r}};\Delta )$, and $D_{\text {eff, Phase}}^y({\textbf {r}};\Delta )$. For reasons described in the preceding text, equation (8) is numerically stable and therefore does not need to be regularised. The subsequent phase extraction, that is equation (9), is ill-posed close to the Fourier-space origin $(k_x, k_y) = (0, 0)$ and hence an appropriate Tikhonov regularisation should be applied following equation (13). For severely ill-posed cases, the phase extraction can be further stabilised by utilising instances of equation (4), substituting in the true effective diffusion coefficient using the method described above, and performing Tikhonov-regularised QR decomposition to solve for $\nabla _{\perp }^{2}\phi _{\text {ob}}({\textbf {r}})$ before applying the inverse Laplacian operator to reconstruct $\phi _{\text {ob}}({\textbf {r}})$.

For the Tikhonov regularisation to operate successfully, $\alpha$ needs to be selected appropriately for the given data. If $\alpha$ is too large, the computed solution will be over-smoothed and will therefore lack fine detail. In the case of image reconstruction, this means the computed solution will have poor spatial resolution, although a high SNR. Conversely, if $\alpha$ is too small, the computed solution will be severely contaminated with errors resulting from numerical instabilities. Evidently, optimising the regularisation parameter is critical to successfully extract multimodal signals, in this case. There are algorithms in the current research literature that optimise the Tikhonov regularisation parameter for a given ill-posed problem, see Park *et al.*^68^ and references therein. However, in image reconstruction, image quality metrics can be used to determine the optimal regularisation parameter. In the present work, we used four metrics:

1. Naturalness Image Quality Evaluator (NIQE)^69^: The NIQE is a blind image quality assessment based on an image’s measurable deviations from statistical regularities observed in natural images, namely a natural scene statistics (NSS) model. A lower NIQE reflects an image with a higher perceived image quality.
2. Azimuthally Averaged Power-Spectrum^70^: The two-dimensional power-spectrum of an image can be calculated by taking the absolute square of the Fourier-transformed image. This can then be azimuthally-averaged, with the centre at the Fourier-space origin $(k_x, k_y) = (0, 0)$, to calculate a one-dimensional power-spectrum that shows the contribution of all spatial frequencies in an image. The noise in the image is reflected by the so-called “noise-floor”, which typically makes up the majority of the signal at high spatial frequencies, and the spatial resolution can be gauged by the “knee” of the power-spectrum, namely the frequency at which noise becomes a significant contribution to the signal.
3. SNR: The SNR measures the magnitude of a signal relative to background noise. It is used to quantify signal quality in an image, and is defined as
  14 $$\begin{aligned} SNR = I_{\text {avg}}/\sigma , \end{aligned}$$
  where $I_{\text {avg}}$ is the signal strength, which can be measured as the average pixel value within a region of approximately uniform signal, and $\sigma$ is the noise as measured by the standard deviation of pixel values. Note that if the noise characteristics are the same inside and outside an object, then it may be easiest to measure $\sigma$ in the region outside the object to avoid variations in the signal that come with a complex object.
4. Human visual perception: Although subjective, meaningful image quality measurements can be made by a human observer’s visual assessment of an image. Typical “desirable” image features for a human observer are sharp edges, fine resolvable sample features, and low noise (locally and globally). A human observer is capable of determining a good compromise between noise and spatial resolution. In this work, the human observers were the four authors, who are all X-ray physicists.

## Applying the theoretical approach to synchrotron SB-PCXI data

To test the proposed MIST approach, we extracted multimodal signals from two samples, a wattle flower (denoted as “wattle” hereafter) and red currant (denoted as “currant” hereafter). These two samples had different X-ray attenuation coefficients, $\beta ({\textbf {r}}')$, and thicknesses, and hence attenuate the X-ray beam differently. In particular, the wattle was weakly-attenuating (almost a pure phase-object) and the currant was non-negligibly-attenuating.

### Experimental procedures

SB-PCXI data of the wattle were collected in experimental hutch 3B of the Imaging and Medical Beamline (IMBL) at the Australian Synchrotron, similar to the setup shown in Fig. 1. The entrance window to hutch 3B was located 135 m from the source. A virtually monochromatic 25 keV X-ray beam was used for imaging with a 100 ms exposure time. The Ruby detector^71^, which has a single pco.edge sensor and lens-coupled scintillator, was positioned $\Delta = 2$ m downstream from the sample. The pixel array was 2560 $\times$ 2160 and a 105 mm macro lens was used to achieve an effective pixel size of 9.9 $\mu$m. The speckle-generating mask was located around 60 cm upstream of the sample, and a combination of grit P40, P80 and P120 sandpapers were simultaneously used. A combination of sandpapers was used in this case such that the generated speckle pattern had a range of high-visibility feature sizes. The speckle-generating sandpaper was placed on a translation motor stage such that it could be brought in and out of the X-ray beam path and also translated transversely to allow the beam to pass through different parts of the mask. The effective speckle-size was 136.4 $\mu$m, as measured by the average full-width at half-maximum of the autocorrelation function^28^ in the horizontal and vertical directions of the reference-speckle field.

The currant sample was imaged using SB-PCXI at the European Synchrotron Radiation Facility (ESRF) beamline BM05; these data were obtained and originally published by Berujon and Ziegler^31^. The setup was similar to that shown in Fig. 1, with an X-ray energy of 17 keV and spectral bandwidth of $\Delta E/E \approx 10^{-4}$, which was produced using a double crystal Si(111) monochromator located 27 m from the X-ray source. The currant was placed on a stage 55 m from the source and images were acquired with 600 ms X-ray exposures. The detector system consisted of a Fast Read-Out Low-Noise (FReLoN) e2V camera coupled to an optical imaging thin scintillator^72,73^. This detector was placed $\Delta = 1$ m downstream from the sample and the effective pixel size of the optical system was 5.8 $\mu$m. The speckle-generating sandpaper with grit size P800 was placed 0.5 m upstream from the sample and had an effective speckle-size of 20.4 $\mu$m.

Image acquisitions for all data used a similar procedure for both samples: dark-current (no X-ray beam) and flat-field (sample and mask not in the beam) exposures were collected before and after the scan, and reference-speckle images with only the speckle mask in the beam were collected before and after the scan. The speckle mask was transversely shifted in the *x*-direction perpendicular to the optical axis (see Fig. 1) to acquire multiple unique sets of SB-PCXI data. To generate a unique set of SB-PCXI data suitable for the present MIST approach the mask should be shifted enough such that a significantly different new reference-speckle pattern, $I_{R}({\textbf {r}})$, is generated – within these experiments we ensured to move the speckle-mask more than ten speckle sizes in one direction. A theoretical minimum of four sets of SB-PCXI data is required for the present approach and the speckle mask does not need to be shifted equidistantly, moreover, it can be shifted to a random mask position to generate each set. Seven and fifteen sets of SB-PCXI data were collected for the currant and wattle, respectively. The collected SB-PCXI data were then processed using a Python script to implement our multimodal signal extraction algorithm. An open-access repository for this script is on GitHub^74^.

### Multimodal signal extraction

Multimodal signals were extracted for the wattle and currant using our new generalised MIST approach. The entirety of the available SB-PCXI data for each sample, that is the maximum number of masks, were used to reconstruct the multimodal signals. Although fewer could be used, this work focuses solely on the new theoretical development; a quantitative analysis of the influence of the number of SB-PCXI data sets is given in Pavlov *et al.*^36^ (see, in particular, Fig. 2 in Ref.^36^). We also note that the computation time does not increase substantially for additional mask positions, with an increase of <5% going from four to fifteen sets of SB-PCXI data for a 2100 $\times$ 2500 pixel image. The established system of linear equations for each sample was solved using the Tikhonov-regularised QR decomposition described above. It was found that the standard deviation of the coefficient matrix divided by $10^{4}$ provided the optimal Tikhonov regularisation parameter for the case of each sample. The phase-object approximation of the sample’s effective diffusion coefficient, $D_{\text {eff, Phase}}({\textbf {r}};\Delta )$, and its spatial derivatives were calculated using this method, from which the true phase-object approximation of the DF signal, $D_{\text {eff, Phase}}^{\text {True}}({\textbf {r}};\Delta )$, was computed. Next, the sample’s phase-shifts, $\phi _{\text {ob}}({\textbf {r}})$, attenuation term, $I_{\text {ob}}({\textbf {r}})$, and true attenuating-object effective diffusion coefficient, $D_{\text {eff, Atten}}^{\text {True}}({\textbf {r}};\Delta )$, were calculated. The calculation of $\phi _{\text {ob}}({\textbf {r}})$ via equation (9) is unstable at the Fourier-space origin; it was regularised using equation (13) with $\alpha = 0.0001$, which was suitable in both cases. The variable $\gamma = \delta /\beta$ was required to calculate the object’s attenuation term, as in equation (12). For the wattle, the generic elemental composition of a plant stem was used within the TS Imaging calculator^75^ to determine its complex refractive index at 25 keV, producing $\gamma _{\,\text {wattle, 25~keV}} = 1403$. For the currant sample, $\gamma$ was taken to be that of water at 17 keV, that is, $\gamma _{\,\text {currant, 17~keV}} = \delta _{\,\text {water, 17~keV}}/\beta _{\,\text {water, 17~keV}} = 1146$.

Multimodal signals were also calculated using our previously-published MIST approaches, that is, equation (6) in Pavlov *et al.*^36^ and equations (16)–(18) in Alloo *et al.*^42^, to provide a point of comparison for the new approach. As described earlier, the approach of Pavlov *et al.*^36^ neglects X-ray attenuation, but Alloo *et al.*^42^ considers it. In both approaches, the effective diffusion coefficient is assumed to be spatially slowly varying. The multimodal signal extraction described by Alloo *et al.*^42^ involved numerical stabilisation via a Tikhonov-regularised “Weighted Determinant” approach. Within this work, the multimodal signals extracted using Pavlov *et al.*’s^36^ algorithm were stabilised in an identical way to that described in Alloo *et al.*^42^, that is a Tikhonov-regularised “Weighted Determinant” approach. The optimal regularisation parameter, in both instances, was equal to the mean of the denominator in equation (23) of Ref. ^42^, divided by 100, for both samples. Here we do not perform a comparison with our directional DF approach^44^.

### Weakly-attenuating sample: wattle flower (wattle)

**Figure 2:**
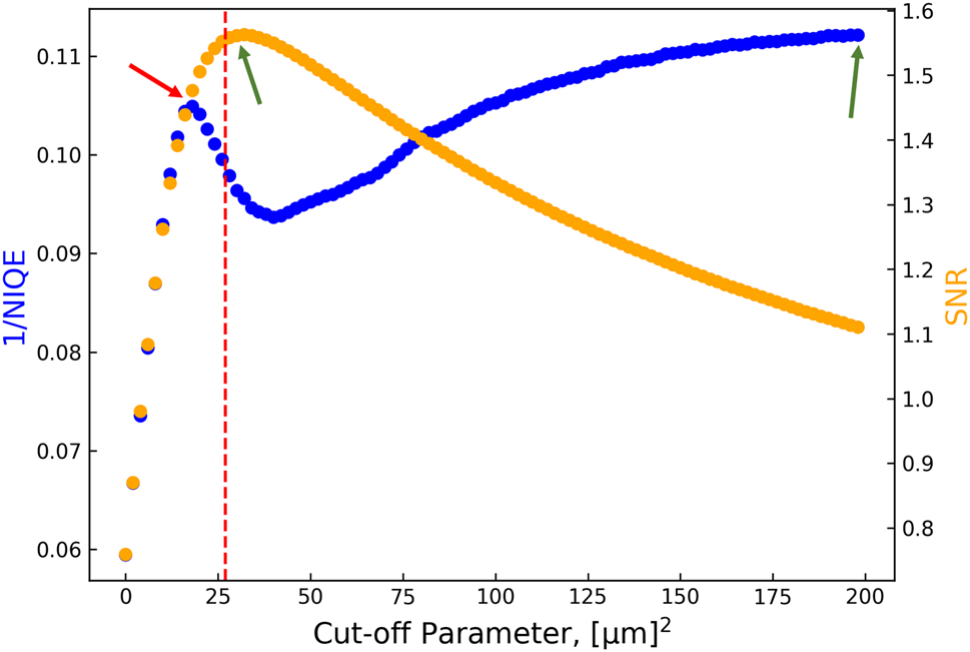
Influence of cut-off parameter, $\rho$, on the image quality of the wattle flower’s reconstructed phase-object approximation of the effective diffusion coefficient, $D_{\text {eff, Phase}}^{\text {True}}({\textbf {r}};\Delta )$. Image quality is measured by the (blue) reciprocal of the Naturalness Image Quality Evaluator (NIQE)^69^ and (orange) signal-to-noise ratio (SNR). The dashed red vertical line denotes the optimal cut-off parameter that appropriately considers both metrics and the verdict of four human observers.

We begin by investigating the wattle sample as this sample conforms most closely to the underlying assumptions of the derived theoretical approach, as it is a weakly-attenuating object. Therefore, the multimodal signals (DF and PC) should be superior using the present approach compared to those extracted using our previous MIST approaches^36,42^. Using the methodology described above, an over-determined system of fifteen linear equations was solved using Tikhonov-regularised QR decomposition, from which the true phase-object DF approximation, $D_{\text {eff, Phase}}^{\text {True}}({\textbf {r}};\Delta )$, attenuation term, $I_{\text {ob}}({\textbf {r}})$, and true attenuating-object DF signals, $D_{\text {eff, Atten}}^{\text {True}}({\textbf {r}};\Delta )$, were calculated. The cut-off parameter, $\rho$, was determined by investigating the SNR and NIQE of the reconstructed $D_{\text {eff, Phase}}^{\text {True}}({\textbf {r}};\Delta )$ for various cut-off parameter values. Figure 2 shows how the value of the cut-off parameter influences the image quality of the reconstructed DF, as measured by the NIQE and SNR of the entire DF reconstruction, where the image noise ($\sigma$ in equation 14) was taken as that in air. As an image with a better-perceived image quality has a lower NIQE, Fig. 2 is shown as the reciprocal of the NIQE such that it follows the same “bigger means better” convention as the SNR shown on the secondary axis. The optimal cut-off parameter is 34 $\mu \text {m}{^2}$ and 198 $\mu \text {m}{^2}$ as measured by the SNR and reciprocal NIQE, respectively, and these are indicated by the green arrows in Fig. 2. As we have described, the NIQE is a blind image quality metric that measures the so-called perceived image quality. Within this work, the NIQE was calculated using an inbuilt function in MATLAB called *niqe(A)*. As per MATLAB’s documentation, *niqe(A)* compares the input image *A* to a default NIQE model computed from images of natural scenes, which follows the original published NIQE approach^69^. Gupta *et al.*^76^ concluded that X-ray images are well-modelled by NSS, which is what MATLAB’s NIQE function uses as its image database. Moreover, NSS models capture the statistical consistencies of X-ray images effectively. This means that the NIQE is a suitable image quality metric to be used in X-ray imaging^77–80^, and specifically, DF imaging. The NIQE score is calculated in regions of high image contrast, and hence, the NIQE model may mistake artefacts for signal. For example, enhanced edges due to residual PC effects, or Fresnel fringes, are considered qualitatively better by the NIQE as they have an increased sharpness and contrast. Such features are present in the reconstructed DF signal when the cut-off parameter is too large, and hence, explains why the NIQE values for these images indicate higher perceived image quality. It follows that the optimal cut-off parameter should be selected by appropriately considering the SNR and NIQE simultaneously. We selected the optimal cut-off parameter to be $\rho =$27 $\mu \text {m}{^2}$ as this balances the local reciprocal NIQE maximum (at approximately $\rho =$18 $\mu \text {m}{^2}$, indicated by the red arrow in Fig. 2), global SNR maximum (at $\rho =$34 $\mu \text {m}{^2}$), and also the human observers’ verdict. Specifically, human observers analysed and scored the reconstructed $D_{\text {eff, Phase}}^{\text {True}}({\textbf {r}};\Delta )$ across the cut-off parameter range of $\rho =$18–34 $\mu \text {m}{^2}$, from which $\rho =$27 $\mu \text {m}{^2}$ was selected to be the best.

**Figure 3:**
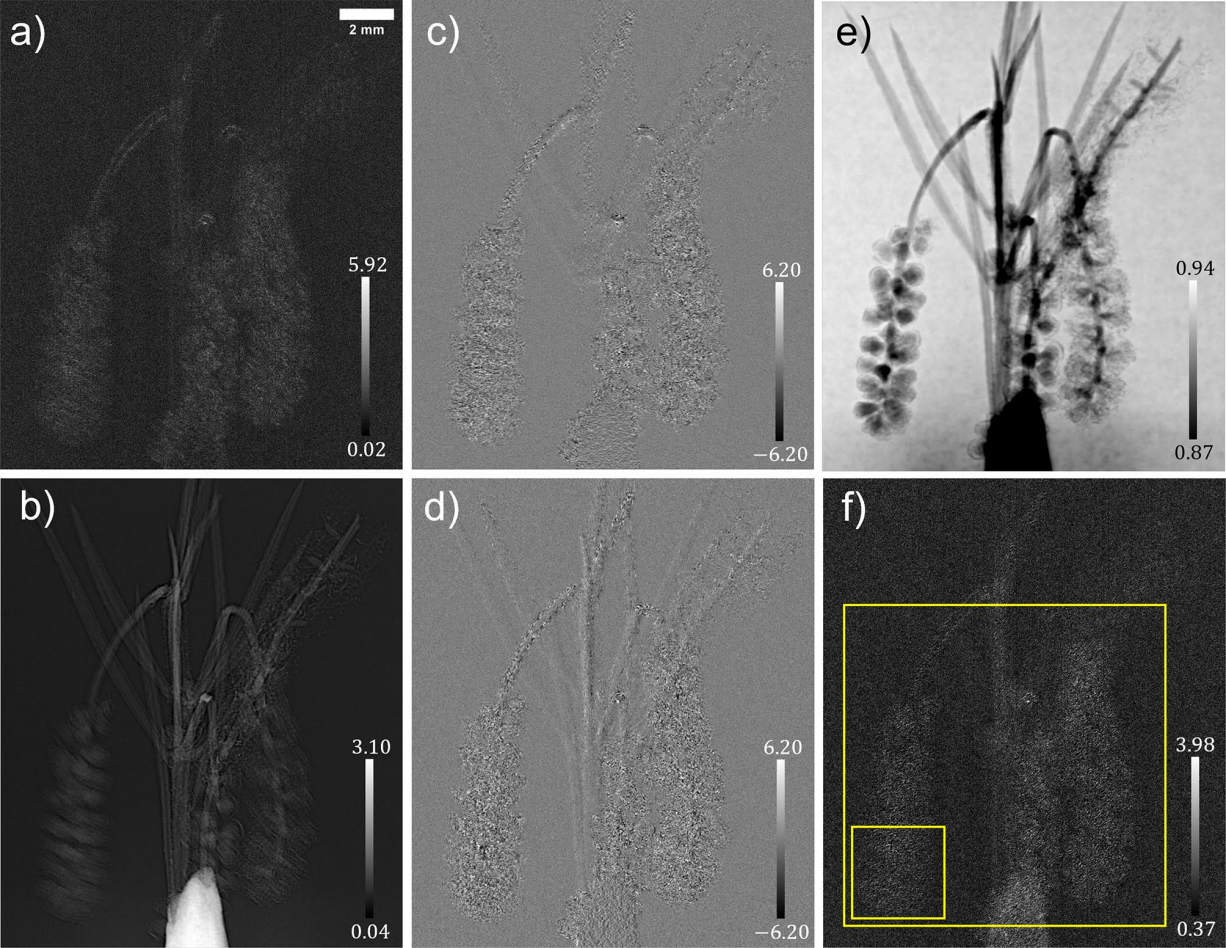
Solutions of the system of linear equations, equation (6), and the wattle flower’s reconstructed multimodal signals; (**a**) is the effective diffusion coefficient, $D_{\text {eff, Phase}}({\textbf {r}};\Delta )$, (**b**) is $\nabla _{\perp }^2\left[ \frac{1}{k}\phi _{\text {ob}}({\textbf {r}})-D_{\text {eff, Phase}}({\textbf {r}};\Delta )\right] _{\text {Recon}}$, (**c**,**d**) are the two spatial derivatives of the effective diffusion coefficient, $D_{\text {eff, Phase}}^y({\textbf {r}};\Delta )$ and $D_{\text {eff, Phase}}^x({\textbf {r}};\Delta )$, respectively, (**e**) is the reconstructed attenuation term, $I_{\text {ob}}({\textbf {r}})$, and (**f**) is the wattle flower’s optimally filtered true effective diffusion coefficient, $D_{\text {eff, Atten}}^{\text {True}}({\textbf {r}};\Delta )$. The greyscale bars in subfigures (**a**,**f**) are $\times 10^{-5}$ $\mu$m, (**b**) is $\times 10^{-7}$ $\mu$m$^{-1}$, and (**c**,**d**) are $\times 10^{-6}$.

**Figure 4:**
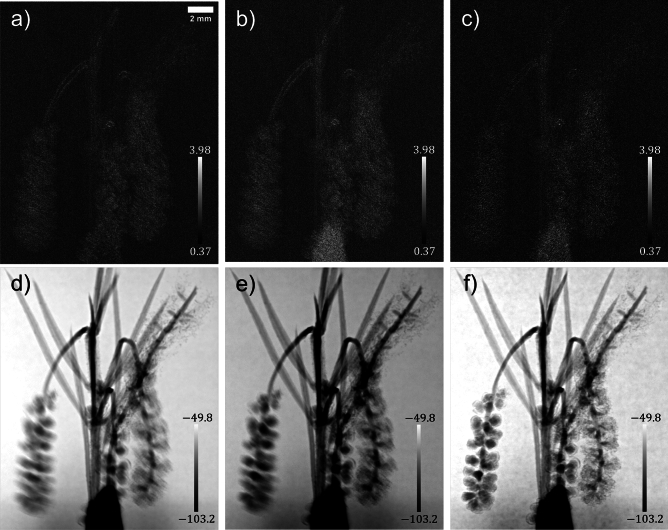
Comparison of wattle-flower multimodal signals extracted using the MIST approaches for rotationally-isotropic diffuse scatter: (**a**–**c**) are reconstructed effective diffusion coefficients, and (**d**–**f**) are phase-shifts (as the wattle flower is a pure phase-object). (**a**,**d**) use Pavlov *et al.*’s^36^ approach, which neglects X-ray attenuation, and (**b**,**e**) use Alloo *et al.*’s^42^ approach, which considers it; both approaches assume the effective diffusion coefficient to be slowly varying. (**c**,**f**) are calculated using the present approach, which considers weak X-ray attenuation and does not approximate the diffusion coefficient as slowly varying. The greyscale bars in (**a**–**c**) are $\times 10^{-5}$ $\mu$m and (**d**–**f**) are $\times 10^{0}$ rads.

Figure 3 shows the computed solutions, $\nabla _{\perp }^2\left[ \frac{1}{k}\phi _{\text {ob}}({\textbf {r}})-D_{\text {eff, Phase}}({\textbf {r}};\Delta )\right]$, $D_{\text {eff, Phase}}({\textbf {r}};\Delta )$, $D_{\text {eff, Phase}}^x({\textbf {r}};\Delta )$, and $D_{\text {eff, Phase}}^y({\textbf {r}};\Delta )$, and the multimodal signals, $I_{\text {ob}}({\textbf {r}})$ and $D_{\text {eff, Atten}}^{\text {True}}({\textbf {r}};\Delta )$, for the wattle sample, where the window and level of each image were set to optimise the respective greyscale range. Although this sample has a weak DF signal, Fig. 3b–d reveal the first- and second-order derivatives of the diffuse scattering signal that had been neglected in prior MIST approaches. It is obvious that the gradient term is stronger at material interfaces on a global and local scale, that is the wattle leaf edges and the filaments that make up each flower, respectively. This follows our initial prediction that the assumption of the DF signal being slowly varying at material interfaces was insufficient; this is furthermore supported in the case of the currant sample which is discussed later. Here, we emphasise that the wattle’s reconstructed $I_{\text {ob}}({\textbf {r}})$ is close to unity, confirming that it is a weakly-attenuating object. Furthermore, in Fig. 3 we show the attenuating-object approximation of the DF signal, however, the reconstructed $I_{\text {ob}}({\textbf {r}})$ demonstrates that the phase-object approximation would be approximately identical to the attenuating-object approximation, given equation (11).

Figure 4 compares the present MIST approach to those in the current MIST research literature^36,42^; the reconstructed DF signals are shown in Fig. 4a–c and the phase-shifts in Fig. 4d–f. Figures 4a,b are qualitatively equivalent with regard to the resolvability of the wattle’s leaves and filaments. Quantitatively the reconstructed DF signal in Fig. 4b is larger than that in Fig. 4a, owing to the former being a phase-object approximation and the latter considering X-ray attenuation, and hence there is a higher reconstructed DF signal in regions that attenuated the X-ray beam more; this agrees with what was found in Alloo *et al.* ^42^ The DF signal computed using the present approach, Fig. 4c, initially appears to have a weaker reconstructed signal within the wattle’s leaves and filaments. However, after inspecting the reconstructed phase-shifts using all MIST approaches, Fig. 4d–f, it becomes apparent the DF signals reconstructed using the alternative approaches have predominant blurring at the wattle’s flowers. Moreover, the wattle’s DF signal from the filaments that make up each flower cannot be well-described by assuming the DF is slowly varying, as assumed in Pavlov *et al.*^36^ and Alloo *et al.*^42^ For this sample, the previous MIST formalisms are unable to detect the rapidly-varying features inside the wattle filaments and its edges. This is apparent in the reconstructions of the phase-shifts and effective diffusion signal. There is also an apparent inhomogeneity in the reconstructed background signal (in air) using the different variants of MIST, that is Pavlov *et al.*^36^, Alloo *et al.*^42^, and that presented here, particularly in the reconstructed phase-shifts. This variation arises from the theoretical assumptions and hence mathematical operations associated with each approach, namely, the sample-specific Fourier-space filtering used in Alloo *et al.*^42^ but not Pavlov *et al.*^36^ or that presented here. This weakly-attenuating sample demonstrates that our new approach gives reconstructed multimodal signals that are qualitatively superior compared to the previously-published MIST approaches. Note that a quantitative comparison of the reconstructed signals’ image quality is provided in a following section.

**Figure 5:**
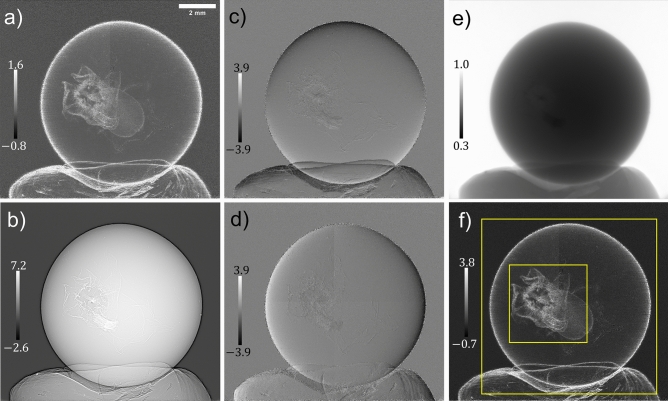
Solutions of the system of linear equations, equation (6), and the red currant’s reconstructed multimodal signals; (**a**) is the effective diffusion coefficient, $D_{\text {eff, Phase}}({\textbf {r}};\Delta )$, (**b**) is $\nabla _{\perp }^2\left[ \frac{1}{k}\phi _{\text {ob}}({\textbf {r}})-D_{\text {eff, Phase}}({\textbf {r}};\Delta )\right] _{\text {Recon}}$, (**c**,**d**) are the two spatial derivatives of the effective diffusion coefficient, $D_{\text {eff, Phase}}^y({\textbf {r}};\Delta )$ and $D_{\text {eff, Phase}}^x({\textbf {r}};\Delta )$, respectively, (**e**) is the reconstructed attenuation term, $I_{\text {ob}}({\textbf {r}})$, and (**f**) is the red currant's optimally filtered true effective diffusion coefficient, $D_{\text {eff, Atten}}^{\text {True}}({\textbf {r}};\Delta )$. The greyscale bars in subfigures (**a**,**f**) are $\times 10^{-5}$ $\mu$m, (**b**) is $\times 10^{-7}$ $\mu$m$^{-1}$, and (**c**,**d**) are $\times 10^{-6}$.

### Attenuating sample: red currant (currant)

We now turn to the non-negligibly-attenuating currant sample, which tests the breadth of applicability of the proposed approach. Using an identical methodology to that described for the wattle, the over-determined system of linear equations for seven sets of SB-PCXI intensity data was solved using Tikhonov-regularised QR decomposition, from which the currant’s true phase-object DF approximation, $D_{\text {eff, Phase}}^{\text {True}}({\textbf {r}};\Delta )$, attenuation term, $I_{\text {ob}}({\textbf {r}})$, and true attenuating-object DF, $D_{\text {eff, Atten}}^{\text {True}}({\textbf {r}};\Delta )$, signals were calculated. The relationship between the reconstructed $D_{\text {eff, Phase}}^{\text {True}}({\textbf {r}};\Delta )$’s NIQE and SNR as a function of cut-off parameter was similar to that shown for the wattle (Fig. 2) sample, and hence, an identical procedure was used to determine the optimal cut-off parameter of $\rho =$ 21 $\mu \text {m}{^2}$.

Figure 5 shows all of the relevant solutions and multimodal signals for the currant sample using the above-described method; Fig. 5a–d shows the reconstructed solutions of the system of linear equations, and Fig. 5e,f displays the currant’s attenuation term and true attenuating-object approximation of its effective diffusion coefficient, respectively. Similar to the case of the wattle, the gradient term is stronger at the periphery of the entire currant sample and at the internal fibres. Figure 5e shows the currant’s attenuation term, where the microstructure is indistinguishable due to its attenuation-based contrast/signal being small. Such microstructure, which is unresolved in the attenuation term, induces measurable SAXS, and hence, can be readily resolved in the reconstructed DF signal shown in Fig. 5f. It is important to make note of the currant’s reconstructed $I_{\text {ob}}({\textbf {r}})$, particularly how far from unity it is. This indicates that the attenuating-object approximation for the effective diffusion coefficient should be used to reconstruct a true DF signal, as shown in Fig. 5f.

**Figure 6:**
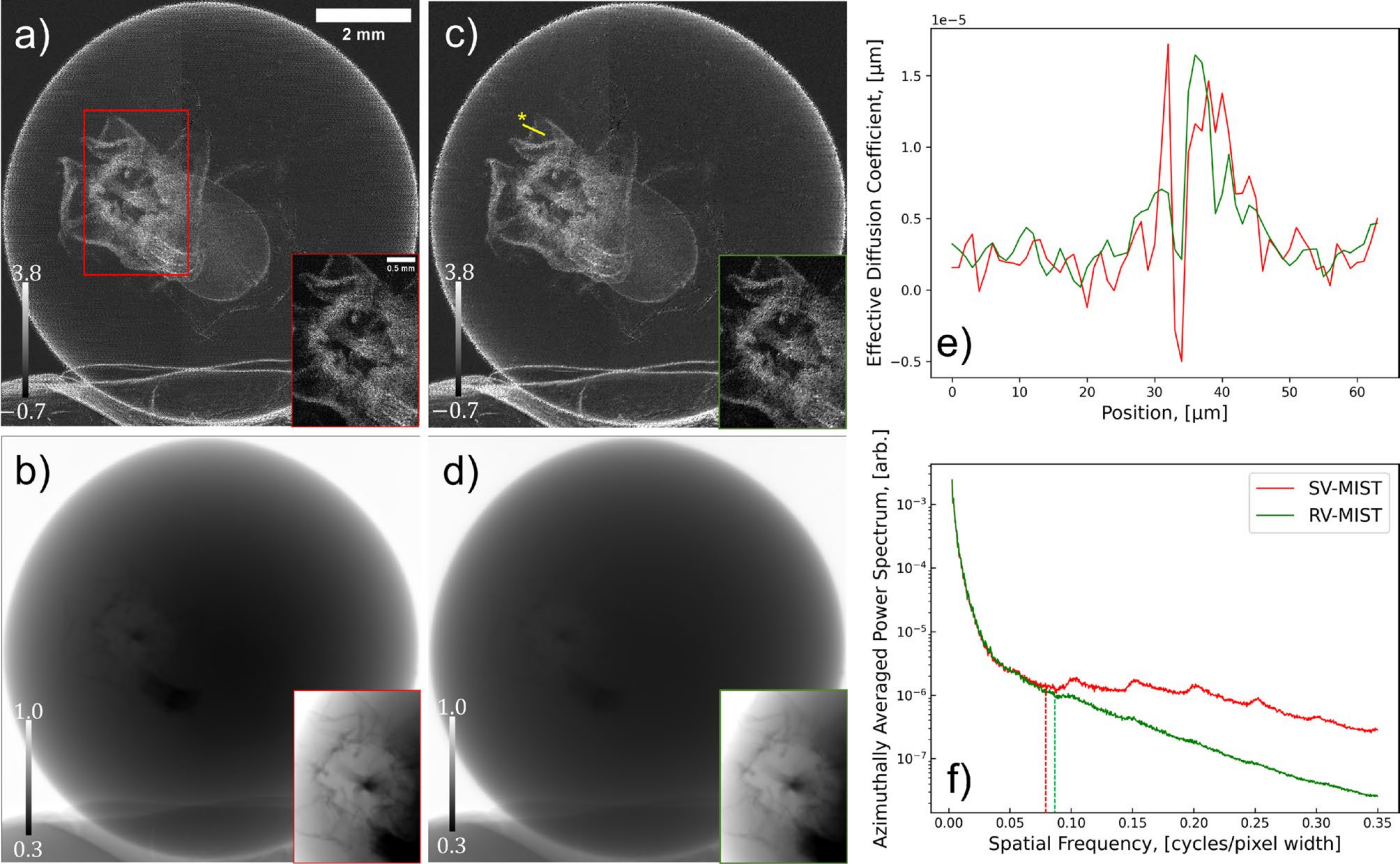
Comparison of the red currant’s multimodal signals extracted using the approach published in Alloo *et al.*^42^, (**a**,**b**), which assume the red currant’s effective diffusion coefficient is slowly varying (SV-MIST), and those presented within this study, (**c**,**d**), which have no assumptions regarding the effective diffusion coefficient (RV-MIST). The effective diffusion coefficient reconstructions are shown in (**a**,**c**), and the currant’s attenuation term is shown in (**b**,**d**). The yellow-asterisk line profile in (**c**) is shown in (**e**), and (**f**) shows the azimuthally averaged power spectra. The vertical dashed lines in (**f**) denote the approximate knee of the respective power spectra. The greyscale bars in (**a**,**c**) are $\times 10^{-5}$ $\mu$m. The displayed greyscale ranges for the subfigures’ magnified regions (**a**–**d**) are [0.01–2.09]$\times 10^{-5}$ $\mu$m, [0.35–0.51], [0.08–1.79]$\times 10^{-5}$ $\mu$m, and [0.38–0.46], respectively.

Figure 6 gives a direct comparison of the currant’s multimodal reconstructions, using (a) the most recently published rotationally-isotropic-scatter “slowly-varying MIST (SV-MIST)” approach^42^ (Fig. 6a,b), and (b) the current “rapidly-varying MIST” (RV-MIST) approach (Fig. 6c,d). Here, we only compare the present approach to the rotationally-isotropic MIST approach^42^ that considers X-ray attenuation, as that published by Pavlov *et al.*^36^ would erroneously reconstruct the currant’s DF signal since the sample significantly attenuates the X-ray beam. Figures 6a,c show a local reconstruction of the currant’s $D_{\text {eff, Atten}}^{\text {True}}({\textbf {r}};\Delta )$ and Fig. 6b,d are the $I_{\text {ob}}({\textbf {r}})$ reconstructions; magnified regions of each extracted signal are also shown. By comparing the reconstructed DF signals, it is evident that the new approach increases spatial resolution, decreases noise, and also provides a greater subject-contrast between unresolved microstructure. Moreover, the small fibrous network surrounding the currant pip, otherwise known as the pericarp, is more resolvable in Fig. 6c than Fig. 6a. A line profile across a single currant fibre, indicated by the yellow asterisk in Fig. 6c, is shown in Fig. 6e. This fine feature is resolvable in the DF image reconstructed using the current approach (denoted by the green trace), but not when the DF is assumed to be slowly varying (denoted by the red trace). When the DF is assumed to be slowly varying, there are supposedly Fresnel fringes, or residual PC, at the boundaries of the traced pericarp fibre in the recovered DF signal; this is the high-low intensity region at approximately 30 $\mu$m and 35 $\mu$m in Fig. 6e. When this assumption is relaxed, the apparent DF signal induced by the strong phase effects is reconstructed appropriately, such that the small fibre is resolvable. This line profile indicates the evident spatial resolution difference between the two images, which is further supported by the azimuthally averaged power spectra in Fig. 6f. These power spectra were calculated in a global region in both DF images which encased the entire currant (the larger yellow box in Fig. 5f). From these power spectra, it is evident that there is a decrease in noise, shown by the reduction in high spatial frequency components (lower noise floor), and an increase in spatial resolution, shown by the higher spatial-frequency position of the power-spectrum knees (denoted by dashed vertical lines), when the DF is considered to be rapidly varying. Although the visibility of the currant’s pip and the pericarp is low in both reconstructions of the attenuation term, Fig. 6b,d, the subject-contrast appears higher when using the previous MIST approach (when the images are shown on the same greyscale range). However, when the greyscale range is optimised independently for each attenuation-term reconstruction, as shown in the magnified regions for each reconstruction, the images look almost identical.

**Figure 7:**
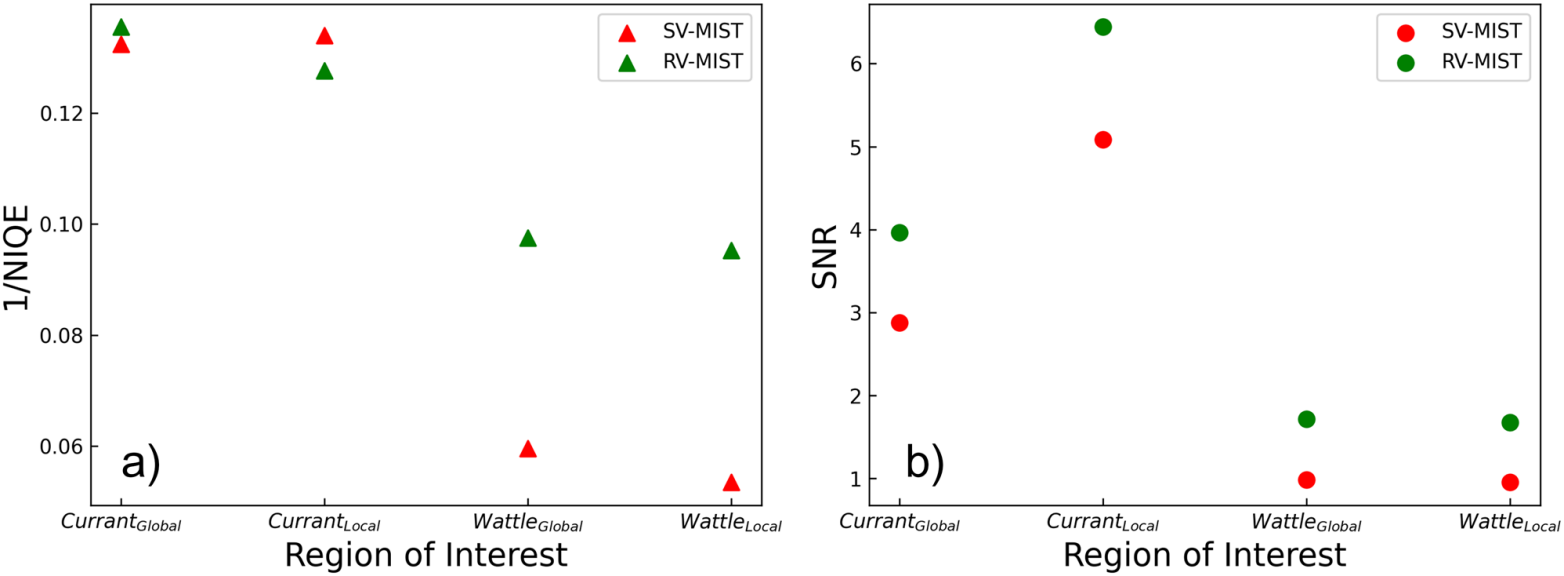
Image quality metrics, (**a**) reciprocal of the NIQE and (**b**) SNR, of the reconstructed effective diffusion coefficients using Alloo *et al.*’s^42^ approach (denoted by the red “SV-MIST” label), and that presented within this study (denoted by the green “RV-MIST” label). Subscript “*Global*” and “*Local*” denote the region of interest used for calculations; these are indicated in Figs. 3f and 5f for the wattle flower (wattle) and red currant (currant), respectively.

## Image quality of reconstructed effective diffusion coefficients

To quantitatively compare the wattle’s and currant’s reconstructed DF signals using the present approach (RV-MIST) and those calculated using the DF slowly-varying attenuating-object approach presented in Alloo *et al.*^42^ (SV-MIST), we investigated the SNR (with $\sigma$ in equation (14) taken as that in air for both samples) and NIQE^69^ for distinct regions. Two regions in each of the reconstructed DF signals were investigated for each sample; the so-called *Global* region which contained the entire sample, and a *Local* region which encompassed only critical structural features of the sample. The *Global* and *Local* regions, denoted by the large and small yellow boxes, respectively, are indicated in Figs. 3f and 5f, for the wattle and currant, respectively. The NIQE and SNR were calculated for both of the reconstructed DF signals in each of the described regions, for each sample. Figure 7 provides a summary of the image quality metrics using the two MIST approaches. The red markers denote the DF signal calculated using the SV-MIST approach^42^ which assumed the DF to be slowly varying, but considers X-ray attenuation, and the green markers denote the new RV-MIST approach which considers rapidly-varying DF behaviour but only weak X-ray attenuation. It is shown from the SNR that the new approach reconstructs superior-quality DF images across all regions in both samples. The reciprocal NIQE values show similar behaviour for the DF signals, with only one datapoint as an exception, where the local reconstruction of the currant is perceived as “better” when the DF is assumed to be slowly varying. This scoring can be explained by the previously-mentioned point describing how the NIQE interprets the residual PC as a sharper edge, due to its increased contrast, when in reality it is incorrectly describing the DF signal. It may be argued that the overall increase in image quality shown in the remaining datapoints is only due to the filtering performed on the new DF reconstruction. However, multiple different filters (e.g., Gaussian and median) were applied to the SV-MIST reconstructions, for which the NIQE and SNR were calculated, and there was no such filtering that gave a comparable image quality to that obtained using the present approach.

The described results for the wattle and currant samples reveal that the present MIST approach reconstructs superior DF signals for weakly- and non-negligibly-attenuating samples, compared to our previously-published MIST approaches. We have also demonstrated that (a) for a weakly-attenuating object the proposed approach gives a qualitatively better attenuation-term reconstruction, and (b) for an attenuating sample both approaches give phase-shift reconstructions with similar image quality. These conclusions were made based on the image quality measures of the reconstructions, rather than the quantitativeness of the reconstructions. Unsurprisingly, the quantitative difference between the reconstructed phase-shift, $\phi _{\text {ob}}({\textbf {r}})$, from the two approaches, is larger for an attenuating object than for a weakly-attenuating object, which is reconstructed equivalently. This is exactly what is expected, based on the underlying assumptions of both theoretical formalisms. That is, the SV-MIST approach^42^ considers X-ray attenuation in the initial Fokker–Planck description, whereas the present approach neglects attenuation initially, before extending to the case of a weakly-attenuating material using the projection approximation. It, therefore, follows that the previous approach more accurately reconstructs the PC signal for an attenuating object, compared to the new approach. The two MIST approaches are complementary methods derived using their own distinct assumptions, therefore it is expected that their respective applicability will be sample dependent. There are regimes in which one approach is more suitable than the other. The present approach is suited to weakly-attenuating objects (e.g., wattle), however, can also be successfully applied to attenuating objects (e.g., currant).

## Concluding remarks

Within this paper, we developed and implemented a rapid deterministic approach that can reconstruct high-resolution multimodal signals of samples using SB-PCXI. The present MIST approach is not restricted to samples that have a slowly-varying DF signal, in contrast to other MIST approaches^36,42,44^, and instead can be used to model rapidly-varying DF behaviour. We applied the approach to two samples that differ in X-ray attenuation and scattering characteristics and compared these signals to those reconstructed using two earlier variants of MIST^36,42^. Using the new approach, the SNR, spatial resolution, and perceived image quality—in the majority of local and global regions-of-interest, across the DF reconstructions for both samples—were higher.

Multimodal X-ray imaging has already proven useful in numerous applications, and this work provides theoretical development towards reconstructing the best possible images using an SB-PCXI technique. It furthermore assists the translation of SB-PCXI into a user-friendly low-dose technique, as the proposed approach theoretically requires just four sets of SB-PCXI data, although more data sets will make the reconstruction more stable (as presented here). It is computationally efficient, requiring just 3 minutes to calculate multimodal signals for a 2100$\times$2500 pixel image using a laptop computer with an 11th Gen Intel(R) Core(TM) i7-1165G7 2.80 GHz processor and 64 GB RAM. Moreover, the experimental setup is simple, and the SB-PCXI technique itself has low coherence requirements.

We anticipate that the present approach can be extended to the case of a highly-attenuating object in which the DF is spatially rapidly varying, and also to the case of rotationally-anisotropic position-dependent SAXS^44^. The directional DF^45,46^ approach using MIST^44^ assumes the DF to be slowly varying. By applying the present approach to solving the directional DF inverse problem, it may be possible to optimise, by means of increased spatial resolution and SNR, the two-dimensional reconstructions, thereby furthering the future goal of MIST tensor-tomography. The present MIST approach could also be used to provide a rapidly-computed deterministic initial guess for alternative speckle-tracking approaches that solve the inverse SB-PCXI problem iteratively^19,31,32,35^. Such iterative techniques—which have a broader domain of applicability because they make fewer assumptions than is the case for our work—are computationally expensive, as the multimodal signals are reconstructed with no definite initial guess. The initialisation of these iterative approaches with the DF signals calculated using the presented MIST approach may help the approaches converge more rapidly to a solution that correctly represents the sample, also making these approaches more appealing for broader adoption.

## Data Availability

The Python script, with appropriate test data, is available in the open-access repository on GitHub^74^. Further experimental data are available upon reasonable request, please contact the corresponding author, S. J. Alloo.
