# Author Correction: M﻿ultimodal intrinsic speckle-tracking (MIST) to extract images of rapidly-varying diffuse X-ray dark-field

**DOI:** 10.1038/s41598-024-57683-x

**Published:** 2024-03-27

**Authors:** Samantha J. Alloo, Kaye S. Morgan, David M. Paganin, Konstantin M. Pavlov

**Affiliations:** 1 School of Physical and Chemical Sciences, University of Canterbury, Christchurch, New Zealand; 2 School of Physics and Astronomy, Monash University, Clayton, VIC Australia; 3 School of Science and Technology, University of New England, Armidale, NSW Australia

Correction to: *Scientific Reports* 10.1038/s41598-023-31574-z, published online 03 April 2023

The original version of this Article contained an error in the Stabilising the SB-PCXI multimodal inverse problem section.

As a result,

“Namely, rather than using QR decomposition to solve the linear system $A\overline{x}=\overline{b}$ for the least-squares solution $\widetilde{x}$, QR decomposition can instead be performed on the system $$(\begin{array}{c}A;\alpha I\end{array})\overline{x}=(\begin{array}{c}\overline{b};0\end{array})$$, where $N\times M$ is the coefficient matrix *A*, “;” denotes a new row, *I* is the $M\times M$ identity matrix, $\alpha$ is the chosen regularisation parameter, and the right-hand-side vector $\overline{b}$ is filled with zeroes to reach the size of $({M}_{N})\times 1$.”

now reads:

“Namely, rather than using QR decomposition to solve the linear system $A\overline{x}=\overline{b}$ for the least-squares solution $\widetilde{x}$, QR decomposition can instead be performed on the system $$(\begin{array}{c}A;\alpha I\end{array})\overline{x}=(\begin{array}{c}\overline{b};0\end{array})$$, where $N\times M$ is the coefficient matrix *A*, “;” denotes a new row, *I* is the $M\times M$ identity matrix, $\alpha$ is the chosen regularisation parameter, and the right-hand-side vector $\overline{b}$ is filled with zeroes to reach the size of $(N+M)\times 1.$”

The original Article has been corrected.

